# Rancid Butter, Cured

**Published:** 1857-12

**Authors:** 


### RANCID BUTTER.

To a pint of water add about thirty drops, that is, about half a teaspoonful, of *Liquor of Chloride of Lime;* wash in this two and a half pounds of insupportably rancid butter; when every particle of the butter has come in contact with the water, let it stand an hour or two, then wash the butter well again in pure water; the butter is then left with the odor, taste, and sweetness of fresh butter. If this is true, it is an important discovery—this preparation of *Lime* having nothing injurious in it.

